# Transcriptomic Responses in the Bloom-Forming Cyanobacterium Microcystis Induced during Exposure to Zooplankton

**DOI:** 10.1128/AEM.02832-16

**Published:** 2017-02-15

**Authors:** Matthew J. Harke, Jennifer G. Jankowiak, Brooke K. Morrell, Christopher J. Gobler

**Affiliations:** a Department of Earth and Environmental Sciences, Lamont-Doherty Earth Observatory, Columbia University, Palisades, New York, USA; b Stony Brook University, School of Marine and Atmospheric Sciences, Southampton, New York, USA; University of Georgia

**Keywords:** microcystis, RNA-Seq, transcriptome, grazing

## Abstract

The bloom-forming, toxic cyanobacterium Microcystis synthesizes multiple secondary metabolites and has been shown to deter zooplankton grazing. However, the biochemical and/or molecular basis by which Microcystis deters zooplankton remains unclear. This global transcriptomic study explored the response of Microcystis to direct and indirect exposures to multiple densities of two cladoceran grazers, Daphnia pulex and D. magna. Higher densities of both daphnids significantly reduced Microcystis cell densities and elicited a stronger transcriptional response in Microcystis. While many putative grazer deterrence genes (encoding microcystin, aeruginosin, cyanopeptolin, and microviridin) were largely unaffected by zooplankton, transcripts for heat shock proteins (*hsp*) increased in abundance. Beyond metabolites and *hsp*, large increases in the abundances of transcripts from photosynthetic processes were observed, evidencing energy acquisition pathways were stimulated by grazing. In addition, transcripts of genes associated with the production of extracellular polysaccharides and gas vesicles significantly increased in abundance. These genes have been associated with colony formation and may have been invoked to deter grazers. Collectively, this study demonstrates that daphnid grazers induce a significant transcriptomic response in Microcystis, suggesting this cyanobacterium upregulates specific biochemical pathways to adapt to predation.

**IMPORTANCE** This work explores the transcriptomic responses of Microcystis aeruginosa following exposure to grazing by two cladocerans, Daphnia magna and D. pulex. Contrary to previous hypotheses, Microcystis did not employ putative grazing deterrent secondary metabolites in response to the cladocerans, suggesting they may have other roles within the cell, such as oxidative stress protection. The transcriptional metabolic signature during intense grazing was largely reflective of a growth and stress response, although increasing abundances of transcripts encoding extracellular polysaccharides and gas vesicles were potentially related to predator avoidance.

## INTRODUCTION

Predator-prey interactions are some of the most important ecological relationships on the planet and often lead to evolving defensive adaptations (1, 2). Phytoplankton have developed a series of defenses against predation, including armor, formation of chains, colonies, spines, swimming, and/or the production of harmful compounds (3, 4). A wide variety of harmful compounds are produced by more than 200 algal species from more than 20 genera to deter grazing or directly kill herbivores (5). In comparison with constitutive defenses, defenses induced by the presence or action of predators may be an effective way to minimize the cost of defense (4). Since zooplankton grazing usually varies both on temporal and spatial scales, the evolution of inducible defenses should be favored over constitutive defenses. In some phytoplankton, zooplankton grazing induces enhanced toxin production (6, 7) or induces the release of volatile chemicals once attacked by zooplankton, which can serve as directional cues by predators of zooplankton, such as fish (8). The presence of grazers can also promote colony formation in some phytoplankton, which reduces grazing pressure by creating a size mismatch (9–11). While the molecular basis for defenses induced by grazers is poorly understood, the use of high-throughput sequencing now makes it feasible to explore such questions in a globally comprehensive manner.

Blooms of toxic cyanobacteria have become a common occurrence in water bodies worldwide, and one of the most pervasive bloom-forming cyanobacteria in freshwater ecosystems is Microcystis (12). Many Microcystis strains produce the potent hepatotoxin microcystin. Thus, persistent blooms of this cyanobacterium pose a risk to those who use impaired water resources for drinking, recreational activities, and fisheries (13). One factor facilitating blooms of Microcystis is its ability to resist and deter zooplankton grazing (14–16). Grazer inhibition by Microcystis has been hypothesized to be related to the synthesis of microcystin (14, 17–19), and Jang et al. (20) demonstrated that Microcystis increases its cellular microcystin content when exposed to zooplankton. However, others have argued that microcystin does not deter grazers. Rantala et al. (21) suggested that the evolution of microcystin synthesis significantly predated that of metazoans and thus proposed the toxin did not evolve as a grazing deterrent. Meta-analyses of laboratory studies have concluded that, although Microcystis reduces zooplankton growth rates, such effects are generally not related to the microcystin content of cultures (15, 22, 23). Within an ecosystem setting, Davis and Gobler (24) quantified grazing rates by multiple classes of zooplankton of microcystin- and nonmicrocystin-synthesizing strains of Microcystis in multiple ecosystems and found that microzooplankton and mesozooplankton grazed toxic and nontoxic strains with similar frequencies and rates.

Beyond microcystin, there is evidence that Microcystis colony formation and synthesis of other secondary metabolites can potentially act as grazing deterrents. Studies have found that larger colonies of Microcystis are poorly grazed, particularly by smaller crustacean zooplankton (15, 25), and Yang et al. (26) reported results from a strain of Microcystis that transformed from unicellular to colonial in direct response to small, flagellated zooplankton grazers that could not consume the colonies. The authors of some studies have concluded the synthesis by Microcystis of protease inhibitors and other metabolites, such as aeruginosin, cyanopeptolin, and microviridin, may both prohibit digestion of cells and discourage zooplankton grazing (4, 27). To our knowledge, no study to date has considered how transcription of genes related to secondary metabolites or other compounds change upon exposure to zooplankton grazers.

This study assessed the global transcriptional response of Microcystis to zooplankton, specifically daphnids. Recent investigations have shown that gene expression in Microcystis is strongly regulated by the nutritional status of the cells (28–32). However, less is known via transcriptomics about the response of Microcystis to grazing. Here, Microcystis was directly and indirectly exposed to the daphnids, Daphnia pulex and D. magna, which were previously shown to be capable of consuming Microcystis (15, 24). After the exposures, the changes in cell densities, microcystin concentrations, and gene expression levels were assessed in Microcystis. Whole transcriptome analyses permitted an investigation of gene pathways associated with the synthesis of known toxins and secondary metabolites, as well as genes not previously identified as related to grazer deterrence.

## RESULTS

### Experimental results.

**FIG 1:**
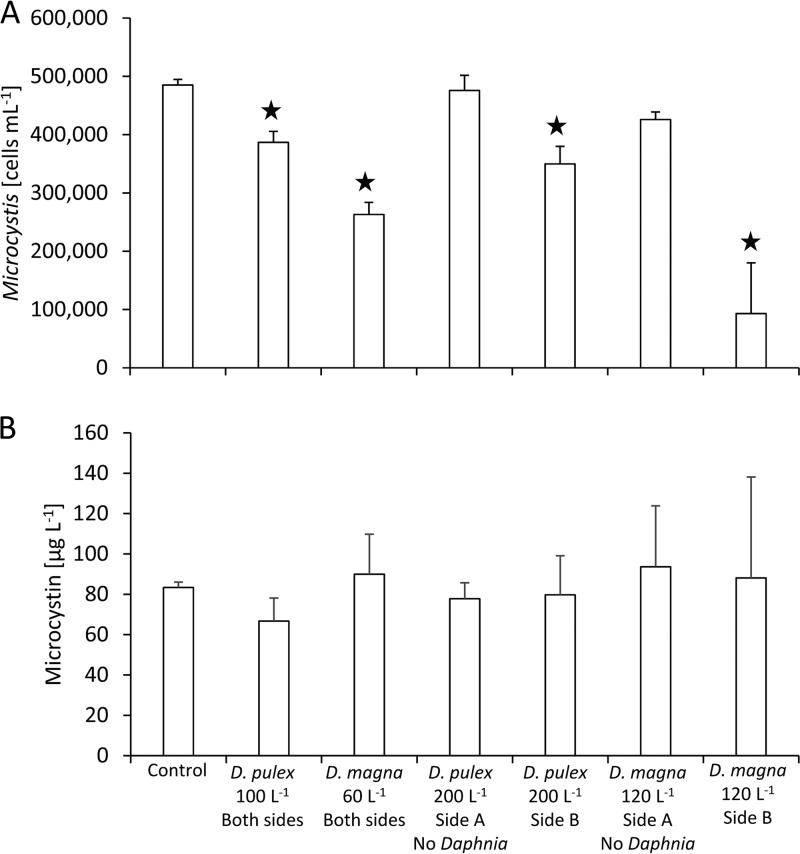
Numbers of Microcystis cells in each chamber (A) and total concentrations of microcystin LR congener equivalents after 24 h (B). For the control, 100 D. pulex liter^−1^, and 60 D. magna liter^−1^, the numbers represent the averages of both sides of the chambers, whereas for the remaining treatments, each side was measured individually for each biological replicate (*n* = 3). Each chamber was seeded with 400,000 cells ml^−1^ at the start of the experiment. ★, *P* < 0.05 versus control, one-way ANOVA.

Microcystis cell densities were significantly reduced after 24 h of exposure to both Daphnia species at both daphnid densities relative to the control (Fig. 1A; one-way analysis of variance [ANOVA], *P* <0.05). The greatest reduction was observed by the treatment with the largest number of D. magna (120 liter^−1^), where the concentration of Microcystis cells was reduced by 80% (Fig. 1A). There was a larger reduction in Microcystis cell densities by D. magna (32 and 78% reduction) compared with those by D. pulex (20 and 26% reduction; Fig. 1A). There was no statistical difference in the total microcystin concentrations among the treatments (Fig. 1B).

### Transcriptomic sequencing.

**FIG 2:**
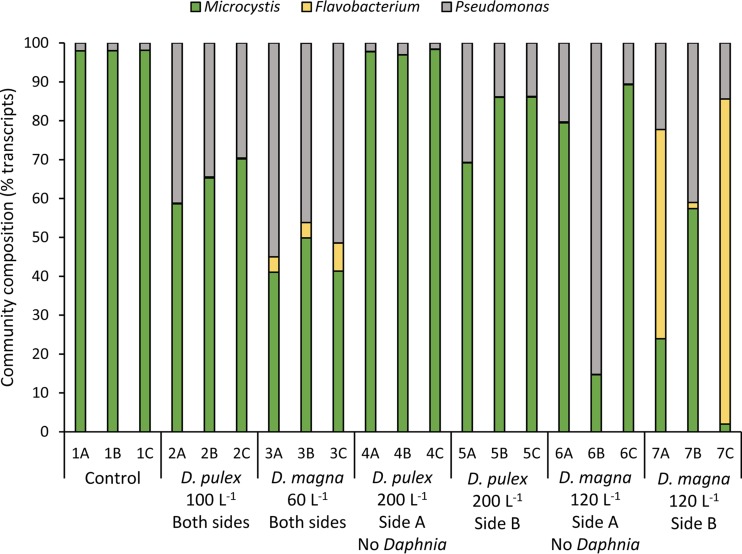
Community compositions at the time the cells were harvested as determined by MetaPhlAn. Bars represent the percentages of reads that mapped to the MetaPhlAn curated marker gene database.

Transcriptomic sequencing yielded, on average, 49 million 100-bp reads per sample (Table 1). Of these, between 2 and 66% mapped to the reference genome (M. aeruginosa NIES-843; Table 1). Taxonomic profiling with MetaPhlAn indicated that more than 98% of mappable reads were assigned to Microcystis in the control treatments, while progressively lower percentages did so in the grazing treatments (Fig. 2). Populations of the Gammaproteobacteria Pseudomonas constituted between 14 and 55% of reads in the chambers with Daphnia present, and a Flavobacterium was in relatively high abundance (54 and 84%) in two of the three chambers for the 120 D. magna liter^−1^ treatment (Fig. 2).

**TABLE 1:** Cell counts, numbers of sequenced reads, and alignment results from RSEM with Bowtie 2^*a*^

Treatment	Sample	Cell counts (cells ml^−1^)	No. of reads			Overall alignment rate (%)
			Total	Not aligned	Aligned	
Control	1A	480,100	51,777,564	30,974,778	20,802,786	40.18
	1B	478,500	42,622,931	24,820,950	17,801,981	41.77
	1C	496,100	43,384,736	26,202,951	17,181,785	39.60
100 D. pulex liter^−1^ (both sides)	2A	397,600	60,070,352	46,457,625	13,612,727	22.66
	2B	397,600	48,893,825	30,650,853	18,242,972	37.31
	2C	365,100	50,960,370	40,117,733	10,842,637	21.28
60 D. magna liter^−1^ (both sides)	3A	267,000	54,632,011	41,514,696	13,117,315	24.01
	3B	281,700	55,558,715	41,630,503	14,010,212	25.07
	3C	240,800	55,613,168	42,396,613	13,216,555	23.77
Indirect, 200 D. pulex liter^−1^ (side A, no Daphnia)	4A	503,200	52,132,318	30,984,344	21,147,974	40.57
	4B	472,100	33,085,445	11,235,493	21,849,952	66.04
	4C	451,700	39,873,147	21,923,667	17,949,480	45.02
200 D. pulex liter^−1^ (side B)	5A	328,400	41,025,237	24,155,144	16,870,093	41.12
	5B	384,200	43,870,155	25,391,764	18,478,391	42.12
	5C	336,400	42,113,885	23,330,398	18,783,487	44.60
Indirect, 120 D. magna liter^−1^ (side A, no Daphnia)	6A	419,100	46,298,279	29,630,922	16,667,357	36.00
	6B	417,400	46,172,455	31,024,552	15,147,903	32.81
	6C	441,000	49,377,092	31,830,973	17,546,119	35.53
120 D. magna liter^−1^ (side B)	7A	54,162	78,193,270	70,356,199	7,837,079	10.02
	7B	192,700	48,819,771	30,788,800	18,030,971	36.93
	7C	32,538	54,691,887	53,595,046	1,096,841	2.01

a Results for each of the sequenced biological replicates (*n* = 3) in the experimental chambers are shown.

### Differential expression upon Daphnia exposure.

**FIG 3:**
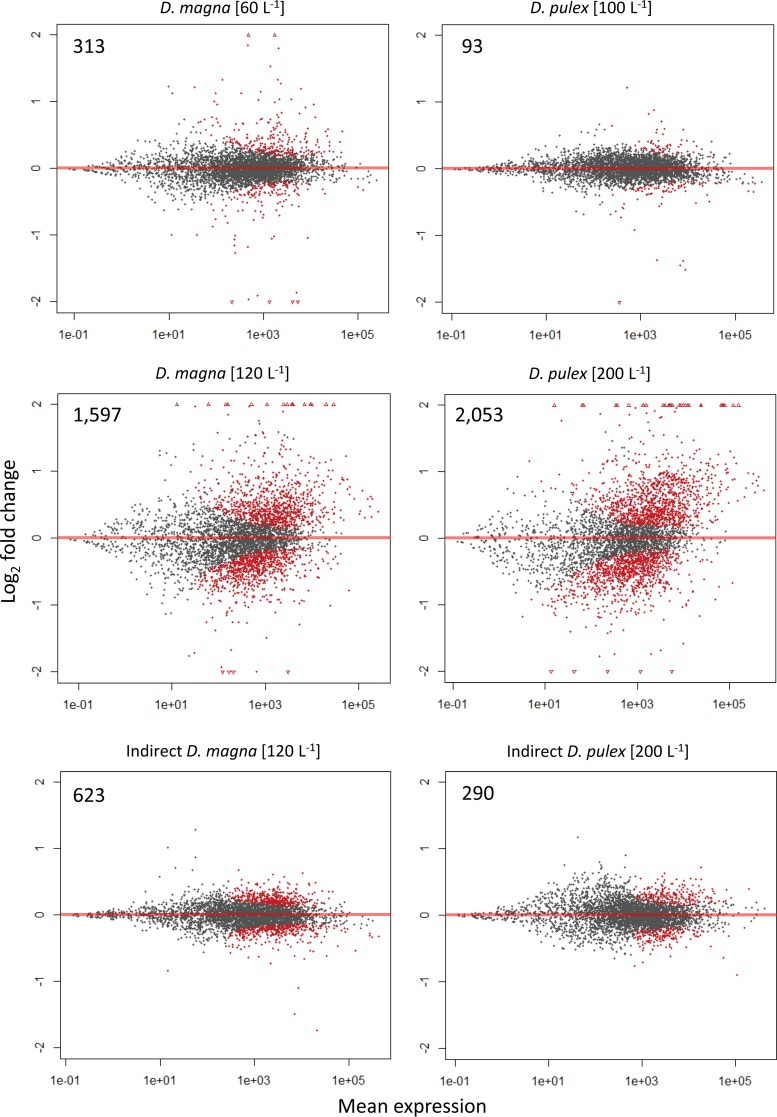
Plots of gene expression versus control for each treatment as determined by DESeq2. Red dots indicate significant differential expression (padj of <0.05). Gray dots indicate no significant expression. “Indirect” denotes responses of M. aeruginosa cells separated from grazers by a 1-μm membrane. All other plots are direct grazer interaction responses. Values in the upper left corners are the numbers of differentially expressed genes (red).

When directly exposed to Daphnia, Microcystis had a larger number of genes differentially expressed at higher concentrations of Daphnia (Fig. 3; see also Fig. S1 in the supplemental material). The strongest response (as measured by fold-change value) was also observed at the highest densities of Daphnia, as treatments with higher densities of each grazer had the largest number of genes with greater than 1 or 2 log_2_-fold change in expression (Fig. 3; see also Fig. S1). Indirect exposure to grazers yielded a more muted differential gene response from Microcystis, with one-third to one-tenth of the number of genes differentially expressed compared with those from direct exposure treatments, and almost all responses were <1 log_2_-fold change (Fig. 3; see also Fig. S1).

**FIG 4:**
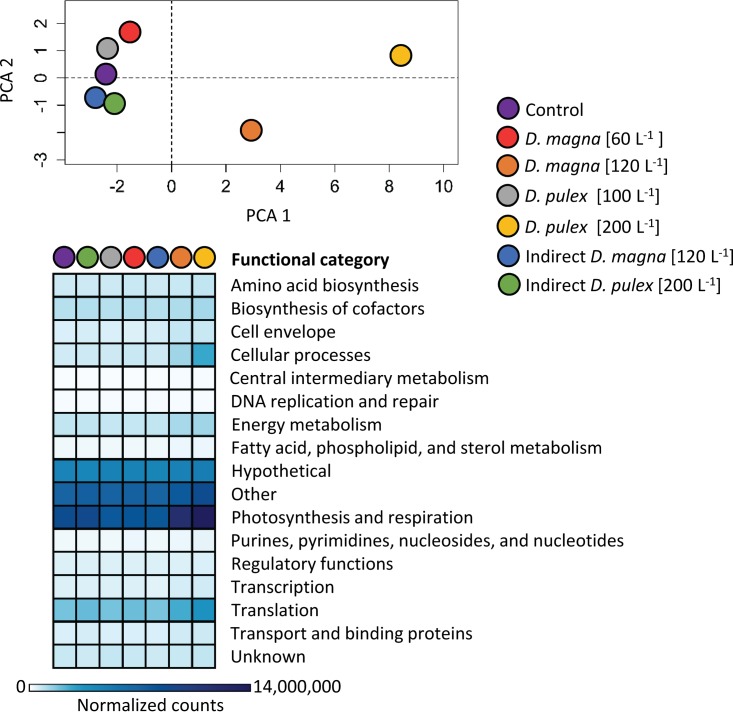
Principal-component analysis and heatmap of functional category signals for each of the treatments. Analysis is based on summed normalized read counts for each functional category as defined by CyanoBase (http://genome.microbedb.jp/cyanobase/GCA_000010625.1).

The majority of transcripts aligned to genes falling within the hypothetical, other, and photosynthesis and respiration functional categories (Fig. 4). Principal-component analysis of all functional category signals suggested that the high grazing treatments (200 D. pulex liter^−1^ and 120 D. magna liter^−1^) yielded larger metabolic responses to grazing/cell lysis than other treatments (Fig. 4). These patterns were largely driven by the abundances of reads assigned to photosynthesis and respiration, cellular processes, and translation functional categories, visualized by the darker colors in the heatmap (Fig. 4).

### Secondary metabolite response.

**FIG 5:**
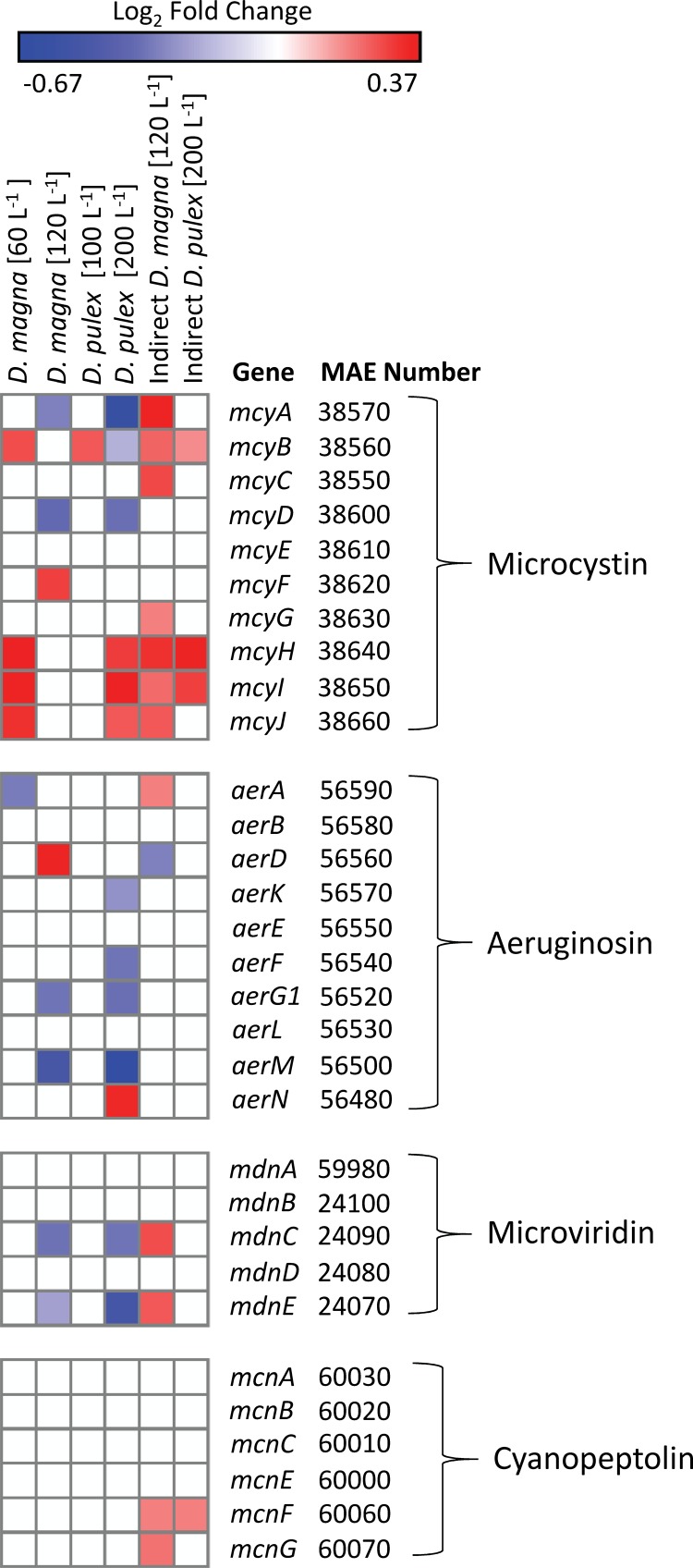
Heatmap of significant gene expression (DESeq2, padj of >0.05) for secondary metabolite gene sets with putative grazer defense association. Blue colors correspond to a decreasing transcript abundance, while red colors correspond to an increasing transcript abundance. White denotes no difference from the control condition.

Of the secondary metabolites surveyed with putative roles in grazer defense, differential gene expression responses were observed with genes encoding microviridin, cyanopeptolin, aeruginosin, and microcystin synthetase. Microcystis cells exposed to low densities of Daphnia (100 D. pulex liter^−1^ and 60 D. magna liter^−1^) and indirectly exposed to daphnids increased their abundances of transcripts of some microcystin peptide synthesis genes (*mcyABC*) and tailoring and transport genes (*mcyHIJ*), while high grazer density treatments (200 D. pulex liter^−1^ and 120 D. magna liter^−1^) decreased the abundances of transcripts for some peptide synthesis genes (*mcyABD*; Fig. 5). The strongest and most consistent responses within the microcystin synthetase cassette were the increasing abundances of transcripts for the tailoring and transport genes (*mcyHIJ*), which increased in 4 of 6 direct and indirect exposures to the daphnids, and *mcyB*, which also increased in 4 of 6 treatments (Fig. 5).

The response for other secondary metabolite genes differed from the patterns displayed by the microcystin synthetase gene cassette. For cyanopeptolin, only two genes (*mcnf* and *mcnG*) were differentially expressed, with increasing transcript abundances observed in both indirect exposure treatments (*mcnF*, 0.19 ± 0.05 log_2_-fold change; *mcnG*, 0.21 ± 0.06 log_2_-fold change; Fig. 5). The genes involved in microviridin synthesis, *mdnC* and *mdnE*, were differentially expressed in three treatments with decreasing transcript abundances during direct contact with Daphnia and increasing transcript abundances when exposed to Daphnia exudates only. Genes encoding aeruginosin synthetase had varied responses. Of the differentially expressed genes in direct grazer responses, the majority displayed decreasing transcript abundances (−0.27 to −0.67 log_2_-fold change), except for *aerD* and *aerN*, which increased (0.35 ± 0.15 and 0.34 ± 0.1 log_2_-fold change, respectively; Fig. 5).

### Other expression responses.

**FIG 6:**
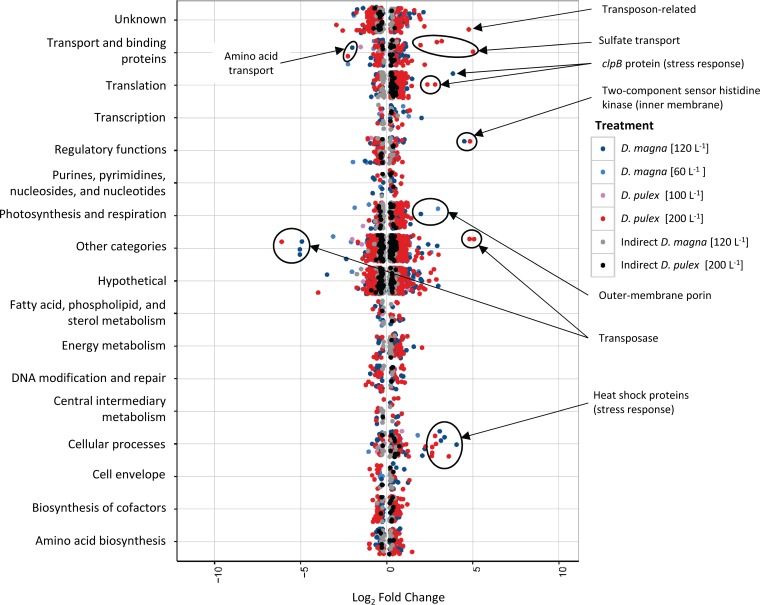
Scatter plot of differentially expressed genes for treatments (relative to the control) as defined by functional categories in CyanoBase (http://genome.microbedb.jp/cyanobase/GCA_000010625.1). Callouts are placed with annotations for some of the highly expressed genes discussed in the text.

Relative to the control, the highest fold-change responses were observed for genes encoding transposases, sulfate transport, and stress response (Fig. 6). Large fold-change responses were mostly observed in the high-density grazer treatments (200 D. pulex liter^−1^ and 120 D. magna liter^−1^; Fig. 6). For example, seven transposases had log_2_-fold change values of >4, of which three had increasing transcript abundances in 200 D. pulex liter^−1^, while three had decreasing transcript abundances in the 120 D. magna liter^−1^ treatment and one decreased by 6.1 ± 0.35 log_2_-fold in the 200 D. pulex liter^−1^ treatment (Fig. 6). For the 200 D. pulex liter^−1^ treatment, there were five sulfate transport genes whose transcripts increased between 2 and 5 log_2_-fold (Fig. 6). In addition, an amino acid transporter (MAE42200) had decreasing transcript abundances in both of these treatments (Fig. 6). With regard to stress response genes, there were 11 genes encoding heat shock proteins that had increasing transcript abundances in 200 D. pulex liter^−1^ and 120 D. magna liter^−1^ treatments, as well as *clpB*, which is involved in translation and encodes a stress-induced multichaperone system (Fig. 6; see also Fig. S2). Lastly, a gene with regulatory functions, encoding a two-component sensor histidine kinase, had fold-change values of >4.5 log_2_-fold in 200 D. pulex liter^−1^ and 120 D. magna liter^−1^ (high grazer density treatments; Fig. 6). Regarding genes putatively associated with extracellular polysaccharide production and export (colony formation defense response), high grazing treatments (120 D. magna liter^−1^ and 200 D. pulex liter^−1^) yielded the greatest number of genes responding. Of the 90 identified extracellular polysaccharide production and export genes, 31 were differentially expressed in 120 D. magna liter^−1^, while 30 were differentially expressed in the 200 D. pulex liter^−1^ treatment (see Fig. S3). Two genes described as outer membrane porins (MAE06090 and MAE27990) were differentially expressed in the high grazing treatments, with increasing transcript abundances of 1.12 ± 0.17 log_2_-fold (see Fig. S3).

Lastly, when observing the overall abundances of read counts (as expressed in variance stabilized transformation of raw counts), the top 20 expressed genes were related to photosynthesis and gas vesicle production (see Fig. S4). Of these, 15 were photosynthesis-related genes, which all had significantly increasing transcript abundances ranging from 0.28 to 1.31 log_2_-fold in the high Daphnia treatments (200 D. pulex liter^−1^ and 120 D. magna liter^−1^; see Fig. S4). For the two genes encoding gas vesicle proteins (MAE37590 and MAE37620), both had increasing transcript abundances in the high grazer treatments (0.36 ± 0.07 and 0.98 ± 0.01 log_2_-fold change in the 120 D. magna liter^−1^ and 200 D. pulex liter^−1^ treatments, respectively) and transcript abundances that increased 0.40 ± 0.08 log_2_-fold in the indirect exposure treatment (passive, 200 D. pulex liter^−1^; see Fig. S4).

## DISCUSSION

During this study, exposure to zooplankton induced a broad and significant transcriptomic response in Microcystis. The abundances of transcripts for several secondary metabolite genes putatively associated with grazing defense, including some, but not all, microcystin synthetase genes, increased with exposure to grazers, while abundances of others did not. Strong and significant differential expression of gene sets associated with stress (such as heat shock proteins), photosynthesis, and gas vesicle production was also observed. There were more genes differentially expressed at the higher densities of each daphnid species, as well as during direct exposure, compared with those during indirect exposure. These patterns give support to the hypotheses that the presence of the grazers induced gene expression in Microcystis and that the expressions of some of these genes were in response to chemicals exuded by the grazing daphnids (indirect exposure), while others were in response to direct exposure and contact with the grazers. Collectively, these findings bring new insight toward understanding the metabolic responses of Microcystis, and perhaps of other cyanobacteria, induced via exposure to herbivores.

The biochemical and ecological roles of the secondary metabolite, microcystin, within Microcystis has long been debated, with previous theories relating microcystin to protection against oxidative stress, quorum sensing, inhibition of competitors, and/or grazing deterrence (33–36), but not as an essential growth factor (37). Synthesis of this compound appears to depend on a sufficient nitrogen supply (29, 30, 38, 39), and there is a growing evidence of the role microcystin plays in protecting cells from oxidative stress (36, 40). In this study, microcystin tailoring and transport genes (*mcyHIJ*) and one peptide synthesis gene (*mcyB*) were the secondary metabolite genes with the most consistent responses to grazers, displaying increasing transcripts in half of direct grazer treatments and in nearly 90% of indirect exposures to daphnids. In stark contrast, the abundances of all other microcystin synthetase genes (*mcyACDEFG*) increased in only 8% of zooplankton treatments. Since all of the peptide synthesis proteins in the microcystin synthetase cassette (*mcyABCDEFG*) must act in unison to make this toxin, our findings suggest *de novo* synthesis of microcystin was not triggered by zooplankton grazing. However, the consistency of the responses for the microcystin tailoring and transport genes (*mcyHIJ*), especially when exposed to zooplankton exudate, is suggestive of a strategy whereby Microcystis purposefully modifies microcystin for various congeners, as has been observed under nitrogen depletion (41). Were this so, it could have implications for cellular toxicity, given the differential potencies of different microcystin congeners (42, 43). The concurrent increases in *mcyH* transcripts putatively involved in the export of microcystin to the periplasmic space of Microcystis (44) suggests zooplankton triggered a purposeful relocation of the microcystin molecule. Given that transcripts of these microcystin tailoring and transport genes (*mcyHIJ*) also increase under limited nitrogen conditions (30), these collective findings suggest that the modification and movement of the microcystin molecule may be a stress response, perhaps supporting the hypothesis that microcystin plays a role as an antioxidant (36, 40), given the concurrent response of multiple stress response genes in these same treatments (e.g., heat shock proteins). This potential use of microcystin for oxidative stress relief is likely linked to the increasing light availability in the high grazer treatments, leading to enhanced photosynthetic production of free radicals. Future transcriptomic studies coupled with proteomics exploring these hypotheses are necessary.

Previous research suggests that Microcystis synthesizes protease inhibitors, such as aeruginosin, cyanopeptolin, and microviridin, to prohibit digestion of cells and discourage zooplankton grazing (4, 27). During this study, the expressions of the 21 genes responsible for synthesizing these three classes of compounds were not highly affected by direct or indirect exposures to daphnid grazers. Half of these genes were not differentially expressed with any treatment, seven displayed mild decreases in transcript abundance in response to at least one of the direct grazer treatments, and five had increases in transcript abundance in response to indirect exposure only. As was the case for microcystin synthetase, since all proteins in the pathway are required to synthesize these compounds, it would appear the zooplankton did not stimulate sudden *de novo* synthesis of these compounds. However, it is possible that some of the genes involved in synthesizing these compounds responded in a time frame that was not considered in this study. For example, Harke and Gobler (30) demonstrated a lag of up to 48 h for the transcription of some genes involved in nutrient acquisition after a sudden change in nutrient concentrations. It is also possible these genes are constitutively expressed. Regardless, the differences in the expression patterns displayed by these secondary metabolite synthesis genes to direct and indirect exposures to zooplankton are consistent with a broader, global transcriptional pattern that emerged during this study as discussed below.

Throughout this study, there were consistent differences between the transcriptional responses to the direct and indirect exposures to daphnids. As described above, the abundances of some secondary metabolite gene transcripts increased when Microcystis was indirectly exposed to grazers, but the abundances of other secondary metabolite genes declined when directly exposed. In contrast, intense direct grazing elicited a broader and deeper transcriptional response from Microcystis, with large increases in transcripts for photosynthesis genes, gas vesicle genes, and heat shock proteins. Such a response suggests cells were seeking to both alleviate cell damage and to increase growth rates via photosynthesis, as well as perhaps increasing the amount of light available by adjusting their vertical position via gas vesicles (45). Alternatively, gas vesicle synthesis might be a means of grazer avoidance, assuming grazers undergo diel vertical migration. Regardless, during catastrophic population losses and potential cellular damage by grazing, cells respond by combating intracellular damage and by ramping up their generation of energy for cellular repair and division via photosynthesis. Under such circumstances, the syntheses of aeruginosin, cyanopeptolin, and microviridin were likely of lesser importance. Thus, several of these genes had decreasing transcript abundances, affirming the role of these compounds as secondary metabolites.

Previous research has demonstrated that Microcystis forms colonies as an inducible defense in response to protozoan grazers and that such colony formation is facilitated by the increased synthesis of extracellular polysaccharides (EPS) (26, 46) and the production of gas vesicles (47). Furthermore, extracellular polysaccharides serve as direct grazing deterrents for some cyanobacteria (48) and eukaryotic algae (49, 50). Studies have also found that increasing levels of extracellular microcystin trigger transcription of polysaccharide biosynthesis-related genes and colony formation in Microcystis spp. (51). During this study, extracellular microcystin levels likely increased in all direct grazing treatments, given that the total microcystin levels did not change but cell densities significantly declined 20 to 80%. In surveying the Microcystis NIES-843 genome, we identified 95 genes potentially involved in EPS production and export. Of these, we observed increasing transcript abundances for genes involved in EPS production (glycosyl transferases and sugar modification enzymes) and EPS transport (outer membrane porins and polysaccharide export proteins; see Fig. S3 in the supplemental material) in the high grazing treatments (120 D. magna liter^−1^, 15 genes and 200 D. pulex liter^−1^, 5 genes), potentially demonstrating attempts by Microcystis to form colonies in response to daphnid grazing, although our experiment was likely too short for this to occur. Further, not all genes binned into this category were differentially expressed, with decreasing transcript abundances for some, indicating that further work is needed to fully characterize the gene pathways necessary for colony formation. Alternatively, the production of extracellular polysaccharides might have directly inhibited the consumption of Microcystis by grazers (48–50).

Heat shock proteins are found in all kingdoms of life, having an important role in cellular defenses to diverse environmental stressors, such as oxidative stress and temperature (52, 53). In Microcystis, it was observed that the heat shock protein genes *hspA* and *htpG* are responsive to temperature (53), while the abundances of transcripts for these and other heat shock proteins (*dnaJ*, *dnaK*, *and grpE*) increase under nitrogen and phosphorus limitation (29, 30). Other heat shock protein genes surveyed by Rhee et al. were not responsive to high temperature, suggesting other stress response roles (53). In Planktothrix agardhii, the abundances of transcripts for *dnaK*, *groEL*, and *groES* (heat shock protein 70 and chaperonin genes, respectively) increased under high light conditions, suggesting roles in oxidative stress defense (54), as oxidative stress often promotes the expression of molecular chaperones and proteases (55). The temperature in this study was maintained at 21°C, demonstrating that the increased expression for this class of genes was unrelated to temperature. Some of these genes may have been transcribed due to the increase in light availability (and associated oxidative stress) from a significant loss of shading by other cells as a result of grazing. Consistent with this hypothesis, the responses of these heat shock proteins may also be related to nonphotochemical quenching (NPQ), a mechanism employed by plants and numerous phytoplankton (including cyanobacteria) to dissipate excess excitation energy as heat (56). Again, given that microcystin may serve as an antioxidant, (36, 40), its modification and intracellular movement, as evidenced by the increasing transcript abundance of *mcyHIJ*, is consistent with the responses of heat shock proteins. Alternatively, given that heat shock proteins have also been implicated in maintaining cellular membranes (57), their transcription might be a repair response by cells exposed to “sloppy feeding” by the daphnids (58), especially if they were of an enzymatic nature and involved in membrane repair. Given the known diversity of heat shock proteins (52, 53) and the large number of genes encoding such proteins present in Microcystis, it is possible that different heat shock proteins were serving different functions when upregulated in response to grazers during this study.

Sulfur plays an important role in photosynthetic organisms, being involved in the biosynthesis of primary and secondary metabolites, coenzymes, and photosynthetic pigments with transport of sulfate linked to cysteine metabolism (59–61). During this study, we observed a number of genes encoding sulfate transport proteins and cysteine-related genes (*cysEHKM*) that had increasing transcript abundances with the D. pulex [200 liter^−1^] treatment. This response was likely related to an increasing cellular demand for sulfur, as we observed increases in transcripts for numerous other gene sets related to growth, photosynthesis, and secondary metabolite synthesis. Furthermore, Microcystis produces dimethyl sulfide (62, 63), and dimethyl sulfide compounds are well-known grazing deterrents (64–66), suggesting some of the enhanced sulfur transport by Microcystis may have been performed in an effort to make sulfonated grazing deterrent compounds, such as dimethyl sulfide (DMS) and dimethylsulfoniopropionate (DMSP).

The abundance of transposase genes in microbial genomes is well known (67), and there is mounting evidence in their role in environmental adaptation (68). During this study, we observed differential expression of multiple transposases and hypothetical genes with transposon-related functions. The strong response of transposases to grazing suggests genome rearrangement may be, to some extant, inducible by grazing pressure or related stressors from grazing. Genome mutations caused by transposases may enable cells to alter physiological responses, facilitating adaptability (69–71) in the face of deleterious environmental conditions, such as nutrient stress (32), viral attack (31), and possibly zooplankton grazing (as in this study).

In conclusion, Microcystis exhibited a transcriptomic response to direct, strong grazing pressure that seemed largely aimed at sustaining vegetative cellular populations. With the reduction in cell densities due to grazing, there was an increased availability of light and associated increases in transcripts for genes involved in photosynthesis and respiration, energy metabolism, biosynthesis of amino acids and cofactors, translation and transcription, and other cellular processes. The expression patterns of genes associated with secondary metabolites were less clear, possibly indicating other roles for these compounds or perhaps their usefulness against other types of microbial interactions. Finally, increases in transcripts of gene sets associated with gas vesicle production and extracellular polysaccharide production may illustrate specific efforts to form colonies and/or avoid zooplankton grazing.

## MATERIALS AND METHODS

### Experimental design.

A transcriptome experiment was conducted to observe global gene expression patterns of Microcystis aeruginosa exposed to daphnid grazers. Experiments were conducted in “exposure chambers” constructed by combining two 250-ml Corning cell culture flasks. A circular 40-mm cutout was made on one side of each culture flask, and a 1-μm pore size, 47-mm polycarbonate filter was glued over the cutout of one flask with aquarium-safe silicone caulk. The flasks were joined together with silicone caulk, creating a novel 500-ml exposure chamber. These chambers enabled the exploration of gene expression changes in Microcystis in response to direct grazing (daphnids in both sides of the chamber) and indirect exposure (daphnids on one side of the chamber only), while allowing dissolved materials (as tested via dye) to uniformly mix between sides when placed on a shaker table for 2 h, but prohibiting Microcystis cells and daphnids from crossing from one flask to the other.

Two cladoceran species were used in the experiments, Daphnia *magna* and Daphnia pulex. Both species were obtained from Aquatic Research Organisms (New Hampshire, USA) from brood EPA OH and were maintained in separate 8-liter aquaria filled with spring water and fed an *ad libitum* diet of Selenastrum capricornutum (∼10^5^ cells ml^−1^) daily. Zooplankton were gently bubbled with air, and aquaria water was exchanged weekly. Prior to the start of the experiments, adult Daphnia were individually picked and placed into spring water for at least 1 h to limit transferring bacteria and food to the experimental chamber. Microcystis aeruginosa clone LE-3 (Lake Erie, USA; 72) was maintained in BG-11 medium illuminated by fluorescent lights that provided a light intensity of ∼100 μmol quanta m^−2^ s^−1^ on a 14:10 light/dark cycle at 21°C.

Treatments included a set of chambers (*n* = 3) containing only Microcystis to serve as a control, a set of chambers (*n* = 3) with D. pulex at 100 liter^−1^ on both chamber sides (direct exposure), a set of chambers (*n* = 3) with D. magna at 60 liter^−1^ on both chamber sides (direct exposure), a set of chambers (*n* = 3) with D. pulex at 200 liter^−1^ on both chamber sides (direct exposure), a set of chambers (*n* = 3) with D. magna at 120 liter^−1^ on both chamber sides (direct exposure), a set of chambers (*n* = 3) with D. pulex at 200 liter^−1^ on one chamber side only (indirect exposure), and a set of chambers (*n* = 3) with D. magna at 120 liter^−1^ on one chamber side only (indirect exposure). Daphnid densities were representative of those previously observed during cyanobacterial blooms (60 to 200 liter^−1^) (73–75). To begin the experiment, all chambers were inoculated with 200 ml of M. aeruginosa LE-3 (log growth phase) at 400,000 cells ml^−1^, and initial samples for cell density determinations and total microcystin concentrations were preserved in Lugol's iodine for quantification and toxin analysis as described below. Daphnia were then transferred into the chambers with a modified transfer pipet. All flasks were placed on a shaker table rotating at a speed of 2 rpm to ensure even distribution of phytoplankton and grazers. After exactly 24 h (to avoid diel changes in gene expression and cell physiology [76]), a second set of samples was obtained for quantifying cell numbers and total microcystin concentrations. Lastly, at the 24-h time point, for transcriptomic sequencing, 100-ml aliquots from each chamber in each treatment were filtered through 0.2-μm Sterivex filters, immediately flash frozen in liquid N, and stored at −80°C. For chambers with Daphnia, care was taken to remove Daphnia with a transfer pipet before filtering.

### Sample analysis.

Lugol's iodine-preserved cells were enumerated using a Beckman Coulter Multisizer 3 Coulter counter with a 50-μm aperture (29). Whole water samples were frozen at −20°C for 24 h, and then the cells were lysed using an Abraxis QuikLyse cell lysis kit. Lysed samples were then analyzed for the hepatotoxin microcystin with a colorimetric immunoassay using an Abraxis microcystins/nodularins (ADDA) enzyme-linked immunosorbent assay (ELISA) kit according to the manufacturer's instructions (77). This analytical precision of this method was ± 2% with a 96 ± 2% recovery of spiked samples. These analyses provided total microcystin measurements, combining extracellular and intracellular fractions. Statistical differences in microcystin concentrations and cell counts between the control and treatments were determined with one-way analyses of variance and *post hoc* multiple comparisons (Holm-Sidak method) using SigmaPlot version 11.0 Build 11.1.0.102, and statistical results were considered against a significance level of α = 0.05.

### RNA isolation and sequencing.

RNA was extracted from triplicate biological samples for each treatment. An RNeasy minikit with RNAprotect bacteria reagent (Qiagen) was used to isolate RNA in a similar manner to that outlined in Ilikchyan et al. (78) with an added 5-min incubation before passing RNAprotect through the filter and with reducing the RNA lysis buffer incubation to 30 min (31). The remainder of the RNeasy minikit protocol was then followed according to the manufacturer's instructions. To remove any traces of contaminating genomic DNA, on-column DNase digestion was performed on RNA samples using RNase-free DNase (Qiagen) according to the manufacturer's instructions. rRNA was removed from total RNA (∼3 μg) using an Epicentre Ribo-Zero magnetic kit (Bacteria) according to the manufacturer's instructions. After rRNA depletion, samples were purified using a Qiagen RNeasy MinElute cleanup kit according to the instructions outlined by the Epicentre Ribo-Zero magnetic kit (Bacteria). The quantity and quality of postdigested RNA were assessed with an Agilent bioanalyzer (RNA integrity of >9.5 for all samples). Samples were stored at −80°C until sequencing.

### Read mapping and analysis.

Prior to read mapping, raw reads were initially characterized with FastQC (http://www.bioinformatics.bbsrc.ac.uk/projects/fastqc/) and were trimmed to remove ambiguous and low-quality reads with Trimmomatic V0.32 (79). Surviving reads were mapped to the reference genome Microcystis aeruginosa NIES-843 (80) using RSEM v1.2.19 (81) with Bowtie 2 (82) with parameters recommend by the RSEM authors. Differential expression between the treatment and reference conditions was computed with DESeq2 using a padj value of >0.95 as the statistical cutoff (83). Briefly, each comparison was input into DESeq2 using the *DESeqDataSetFromMatrix* command with the ∼condition design formula. Differential expression was then analyzed with the *DESeq* function, which first estimates size factors to normalize the data set by library size, and then estimates dispersion by gene and conducts negative binomial GLM fitting and Wald statistics (83). Taxonomic profiling of each sequenced biological replicate was performed with MetaPhlAn v1.7.7 (84), which maps raw sequence reads to a database of predefined clade-specific microbial marker genes. Differentially expressed genes were assigned functional categories based upon categories found in CyanoBase for the Microcystis NIES-843 genome (http://genome.microbedb.jp/cyanobase/GCA_000010625.1). Full differential expression results can be found in Table S1 to S6 in the supplemental material. Principal-component analysis (PCA) for functional category signals was performed from variance-stabilized normalization of reads (DESeq2 function *varianceStabilizatingTransformation*) binned by functional category and visualized using the FactoMineR package in R (85). Heatmaps of these data were visualized with GENE-E (http://www.broadinstitute.org/cancer/software/GENE-E/). The blastp suite (http://blast.ncbi.nlm.nih.gov) was used to elucidate putative functions of hypothetical genes using an E value cutoff of 1e^−5^.

### Accession number(s).

The Illumina sequences reported in this paper have been deposited in the National Center for Biotechnology Information's Sequence Read Archive (accession no. SRP078497).

## Supplementary Material

Supplemental material
