# Supplementary material for: Transcriptomic Responses in the Bloom-Forming Cyanobacterium Microcystis Induced during Exposure to Zooplankton
Source: Appl Environ Microbiol. 2017 Feb 15;83(5):e02832-16. doi: 10.1128/AEM.02832-16 (PMC5311399; doi:10.1128/AEM.02832-16)
Supplement: Supplemental material [file supp_83_5_e02832-16__index.html]

Supplemental material 

# Transcriptomic Responses in the Bloom-Forming Cyanobacterium Microcystis Induced during Exposure to Zooplankton

## Supplemental material

- Supplemental file 1 -

  Distribution of differentially expressed genes for each treatment relative to the control condition (Fig. S1); cumulative expression tree map for the 19 genes identified as heat shock proteins and chaperonins within *Microcystis* (Fig. S2); bar plot and heat map of gene expression for genes potentially involved in extracellular polysaccharide export (Fig. S3); heat map of 20 genes with the most abundant transcripts, expressed as fold change relative to the control (Fig. S4).

  PDF, 692K
- Supplemental file 2 -

  Differential expression results for the *Daphnia magna* 60 liter-1 (Table S1) and 120 liter-1 (Table S2) treatments relative to the control treatment; differential expression results for the *Daphnia pulex* 100 liter-1 (Table S3) and 200 liter-1 (Table S4) treatments relative to the control treatment; differential expression results for the indirect exposure to *Daphnia magna* 120 liter-1 (Table S5) and *Daphnia pulex* 200 liter-1 (Table S6) treatments relative to the control treatment.

  XLSX, 539K
