# Supplementary material for: Transcriptomic Responses in the Bloom-Forming Cyanobacterium Microcystis Induced during Exposure to Zooplankton
Source: Appl Environ Microbiol. 2017 Feb 15;83(5):e02832-16. doi: 10.1128/AEM.02832-16 (PMC5311399; doi:10.1128/AEM.02832-16)
Supplement: Supplemental material [file AEM.02832-16_zam999117696s1.pdf]

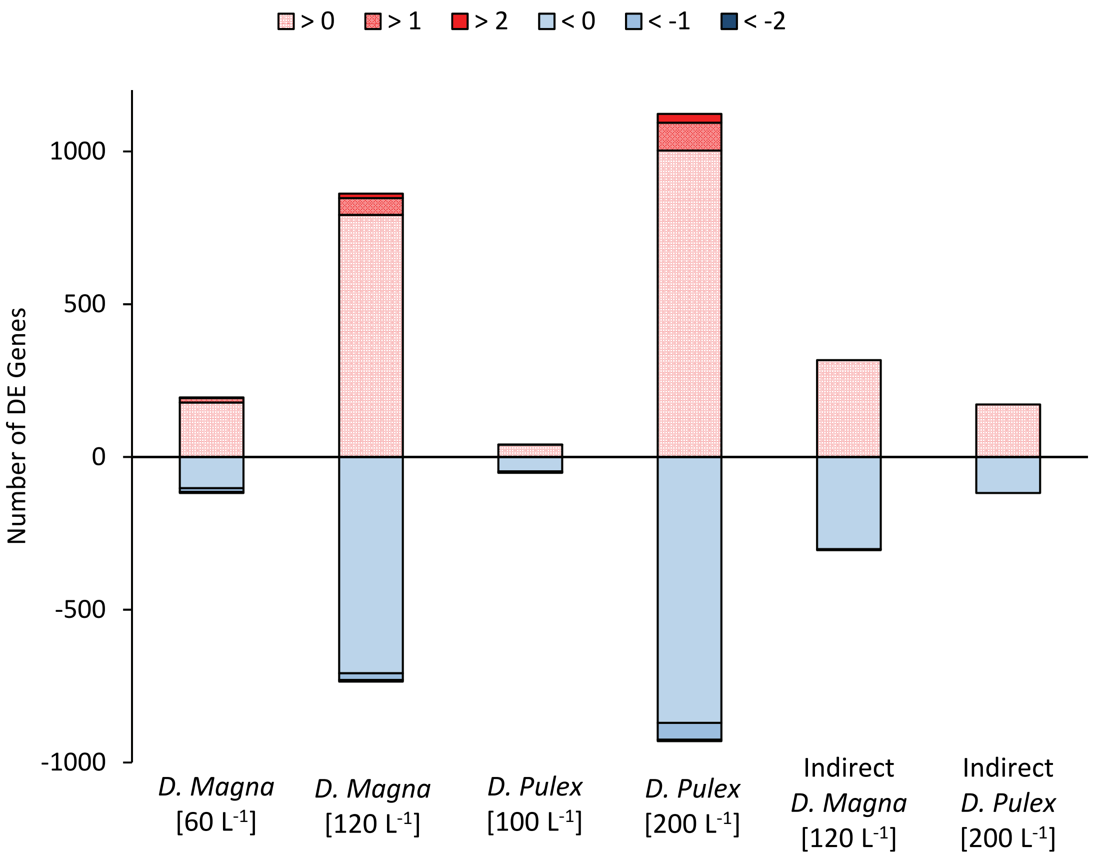

	D. Magna [60 L^{-1}]	*D. Magna* [120 L^{-1}]	*D. Pulex* [100 L^{-1}]	*D. Pulex* [200 L^{-1}]	Indirect *D. Magna* [120 L^{-1}]	Indirect *D. Pulex* [200 L^{-1}]
< -2	4	5	1	5	0	0
< -1	12	22	4	55	3	0
< 0	102	708	47	870	302	118
> 2	2	15	0	29	0	0
> 1	15	55	1	91	0	0
> 0	178	792	40	1003	317	172

Figure S1 The distribution of differentially expressed genes for each treatment relative to the control condition (DESeq2, padj > 0.05). Stacked columns are divided by the distribution of log_2 fold change values as indicated by increasing darkness of color. The table below the figure details this distribution of log_2 fold change values.

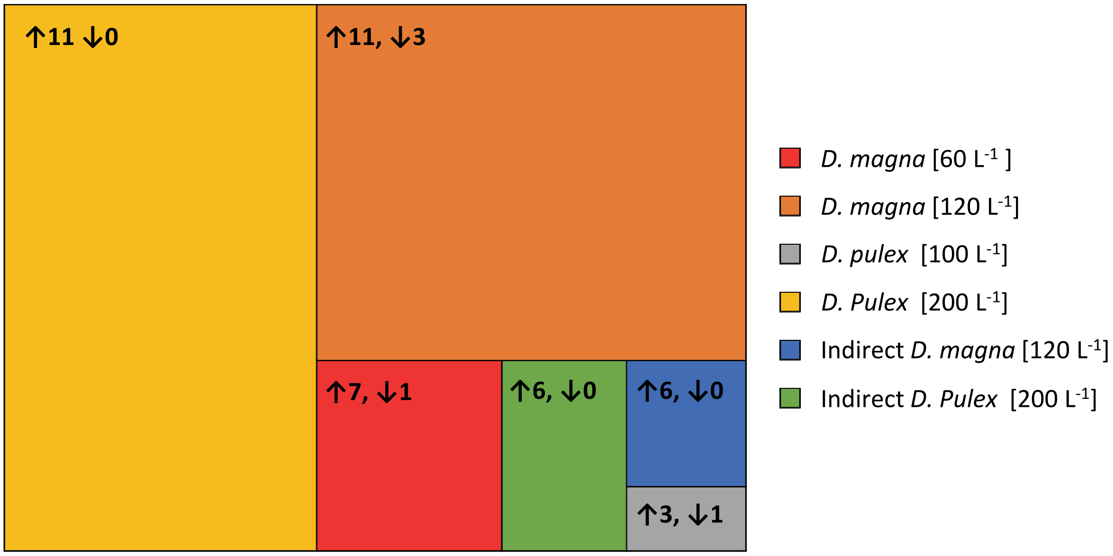

Figure S2 Cumulative expression treemap for the 19 genes identified as heat shock proteins and chaperonins within *Microcystis*. Large boxes equate to greater cumulative expression of these genes. Numbers in the box indicate the number of genes with transcript increases (↑) and decreases (↓).

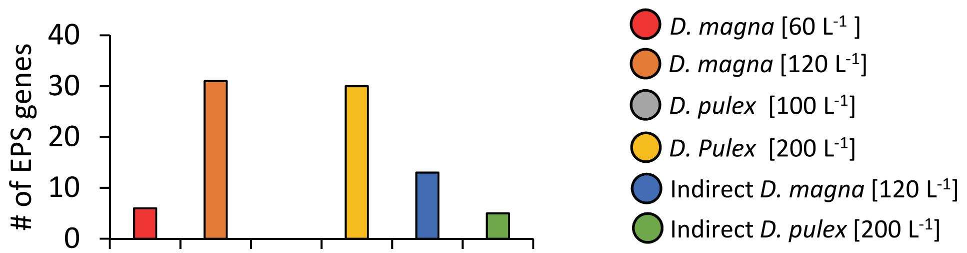

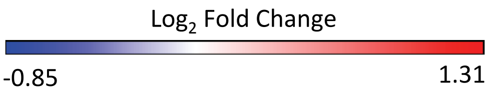

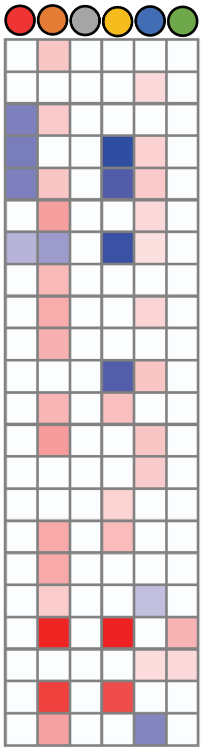

Gene	MAE Number	Product
	42480	Polysaccharide export ABC-2 transport
tagH	42490	Polysaccharide export ABC-transporter
rfbB	57590	dTDP-glucose 4,6 dehydratase
rfbC	180	dTDP-4-dehydrorhamnose 3,5-epimerase
rfbD	190	dTDP-6-deoxy-L-mannose-dehydrogenase
rfbB	18140	dTDP-glucose 4,6 dehydratase
	9020	Probable glycosyl transferase
	58950	Probable glycosyl transferase
	13030	Glycosyl transferase
	42020	Glycosyl transferase family 2
	46400	Glycosyl transferase family 2
	53190	Glycosyl transferase family 2
	46420	Glycosyl transferase family 39
	53110	Glycosyl transferase family 39
	17770	Glycosyl transferase group 1
	24030	Putative O-antigen polymerase
	41410	Putative capsule polysaccharide export precursor
	37150	Polysaccharide export protein
	6090	Porin type major outer membrane protein
	10010	Porin type major outer membrane protein
	27990	Porin type major outer membrane protein
	56470	Porin type major outer membrane protein

Figure S3 Bar plot and heat map of gene expression for genes potentially involved in extracellular polysaccharide (EPS) export. Bar plot denotes the number of genes differentially expressed in this category per treatment while the heat map highlights those genes which had increased transcript abundance in one or more treatments. Blue colors correspond to a decrease in transcript abundance while red colors correspond to an increase in transcript abundance. White denotes no difference from the control condition.

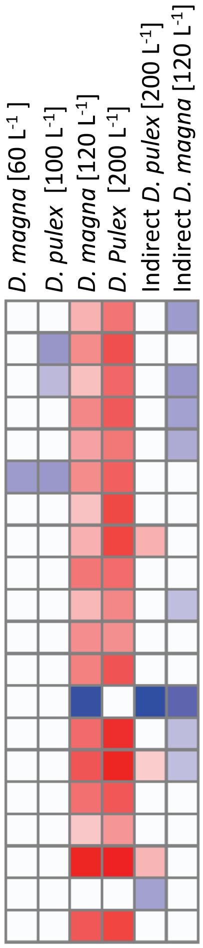

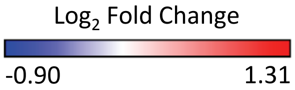

Gene	MAE Number	Product	Function
psaA	45560	P700 apoprotein subunit 1a	Photosynthesis
psbA5	58140	Photosystem II D1 protein	Photosynthesis
cpcA1	24460	Phycocyanin alpha subunit	Photosynthesis
psaB	47570	P700 apoprotein subunit 1b	Photosynthesis
cpcB1	24450	Phycocyanin beta subunit	Photosynthesis
psbA1	10220	Photosystem II D1 protein	Photosynthesis
gvpA1	37590	Gas vesicle protein	Gas vesicle
gvpC	37620	Gas vesicle structural protein	Gas vesicle
psbA3	10510	Photosystem II D1 protein	Photosynthesis
apcB	10260	Allophycocyanin beta subunit	Photosynthesis
apcA	10270	Allophycocyanin alpha subunit	Photosynthesis
psbB	32990	Photosytem II core light harvesting	Photosynthesis
	26790	Hypothetical protein	Unknown
rbcL	47890	Ribulose bisphosphate carboxylase large subunit	Photosynthesis
psbC	41150	Photosystem II CP43 protein	Photosynthesis
apcE	49370	Pycobilisome core-membrane linker protein	Photosynthesis
	39240	MG protoporphyrin IX monomethyl ester cyclase	Photosynthesis
	6090	Portin-type major outer membrane protein	Carbohydrate uptake
	14400	Hypothetical protein	Unknown
atpB	920	ATP synthase beta subunit	Photosynthesis

Figure S4 Heatmap of 20 genes with the most abundant transcripts (variance stabilizing transformation of raw counts) expressed as fold change relative to the control (DESeq2, padj > 0.95). Blue colors correspond to a decrease in transcript abundance while red colors correspond to an increase in transcript abundance. White denotes no difference from the control condition.
